# Observational evidence for groundwater influence on crop yields in the United States

**DOI:** 10.1073/pnas.2400085121

**Published:** 2024-08-26

**Authors:** Jillian M. Deines, Sotirios V. Archontoulis, Isaiah Huber, David B. Lobell

**Affiliations:** ^a^Department of Earth System Science, Center on Food Security and the Environment, Stanford University, Stanford, CA 94305; ^b^Earth Systems Predictability and Resiliency Group, Pacific Northwest National Laboratory, Richland, WA 99354; ^c^Department of Agronomy, Iowa State University, Ames, IA 50011

**Keywords:** groundwater subsidy, water table, maize yield, US Corn Belt, crop model

## Abstract

As climate change shifts crop exposure to dry and wet extremes, a better understanding of factors governing crop response is needed. Recent studies identified shallow groundwater—groundwater within or near the crop rooting zone—as influential, yet existing evidence is largely based on theoretical crop model simulations, indirect or static groundwater data, or small-scale field studies. Here, we use observational satellite yield data and dynamic water table simulations from 1999 to 2018 to provide field-scale evidence for shallow groundwater effects on maize yields across the United States Corn Belt. We identify three lines of evidence supporting groundwater influence: 1) crop model simulations better match observed yields after improvements in groundwater representation; 2) machine learning analysis of observed yields and modeled groundwater levels reveals a subsidy zone between 1.1 and 2.5 m depths, with yield penalties at shallower depths and no effect at deeper depths; and 3) locations with groundwater typically in the subsidy zone display higher yield stability across time. We estimate an average 3.4% yield increase when groundwater levels are at optimum depth, and this effect roughly doubles in dry conditions. Groundwater yield subsidies occur ~35% of years on average across locations, with 75% of the region benefitting in at least 10% of years. Overall, we estimate that groundwater-yield interactions had a net monetary contribution of approximately $10 billion from 1999 to 2018. This study provides empirical evidence for region-wide groundwater yield impacts and further underlines the need for better quantification of groundwater levels and their dynamic responses to short- and long-term weather conditions.

## Significance

The US Corn Belt is a major global producer of both maize and soybeans, with maize output alone worth more than $30 billion per year. Hydrological extremes—both dry and wet—can greatly reduce yields, although most prior work has focused solely on near-surface soil moisture. Here, we demonstrate with region-wide observational data that accessible groundwater increases yield stability over time by providing supplemental water, especially in years with below-average rainfall. Our results suggest that groundwater yield contribution is likely more modest (up to ~7% in dry conditions) than previous work relying on idealistic crop simulations suggests (up to ~24% in dry conditions). Properly understanding and accounting for groundwater’s role will inform climate adaptation in the region.

In rainfed agricultural systems, crop yield potential is often limited by water stress. Conditions with insufficient water to meet crop needs tend to decrease yields (1–3), particularly during sensitive phenological stages (4, 5). At the other extreme, excess soil moisture can cause comparable yield losses due to delayed planting or harvest, oxygen stress, and nutrient loss (6–8) and, in some cases, prevent planting entirely (9). In the United States, drought and excess rainfall are the largest causes of crop loss, together accounting for over 70% of insurance indemnities between 2001 and 2016 (10). As climate change shifts the exposure of crops to both dry and wet extremes, a better understanding of subsurface hydrology’s role in crop performance is needed (3, 11–14).

Recent studies have highlighted the role that shallow groundwater may play in mediating impacts from water stress in agricultural systems. Globally, the water table is estimated to be within or near the root zone on up to 17% of land area (15) and, in natural systems, can supplement plant water use either directly via root uptake or indirectly through increased soil moisture resulting from capillary action. These phenomena are collectively referred to as the “groundwater subsidy” (16–18). Zipper et al. (19) introduced a modified concept for agroecosystems termed the “groundwater yield subsidy,” defined as the increase in crop yields resulting from both direct and indirect groundwater contributions compared to free drainage conditions where the water table is too deep to influence yield outcomes. Thus, opportunities for groundwater yield subsidies arise when yield-limiting climate water deficits occur in the presence of plant-accessible groundwater, particularly during key growth stages. Conversely, when very shallow groundwater levels contribute to water logging, this leads to a negative yield subsidy or “groundwater yield penalty” (19).

Groundwater subsidies and penalties have been documented in small-scale field studies able to directly monitor groundwater levels and yields while controlling for other factors (19–22). Crop-hydrology simulation models have further demonstrated groundwater influence and can isolate yield effects from changing groundwater levels (11, 23–26), but the relevance of these results to real-world agricultural landscapes remains unclear. Regional-scale empirical impacts are hard to assess due to the difficulty of adequately characterizing spatiotemporally variable groundwater levels, soil properties, and management conditions (i.e., tile drainage) and can be obscured by the averaging effects that occur when using regional yield statistics (7, 27). Still, work to date has found evidence of groundwater influence based on aggregated statistics combined with static groundwater maps (28), sparse well data (29), and proxies for groundwater presence such as topographic features (13, 30) or the presence of tile drainage systems (25).

In the US Corn Belt, agriculture is primarily rainfed and water tables are shallow (15, 31), prompting widespread installation of tile drainage approximately 0.8 to 1.2 m beneath the surface to lower the water table and enable successful crop growth (32). With typical rooting depths of maize, a dominant crop, ranging from ~90 to 150 cm (33, 34), groundwater could contribute water through both direct root uptake and enhanced soil moisture through capillary rise. Indeed, there is growing evidence of regional groundwater yield impacts. Analyses of county yield statistics found reduced drought sensitivity and increased sensitivity to excessive rainfall when groundwater was present (3, 7, 28). Rizzo et al. (25) developed regional crop simulations with and without water limitations, finding that unlimited water (their approximation for accessible groundwater) reduced interannual yield variability and accounted for 6% of rainfed maize production overall, and 24% in years with severe drought. They then compared simulation outcomes with observed county yields, finding counties with greater proportions of tile drainage—an indicator of shallow groundwater presence—better matched simulations with unlimited water. Notably, they suggested that ~40% of rainfed maize area in the Corn Belt is influenced by groundwater.

While these regional findings demonstrate a strong need to account for groundwater in the US Corn Belt, they are limited by coarse yield data and proxy groundwater data that do not vary with time. Field scale work using lysimeters finds that groundwater yield contributions vary with water-table depth (21) and groundwater levels respond to both short- and long-term trends in climate, groundwater extraction, and land use (29, 35).

**Fig. 1.**
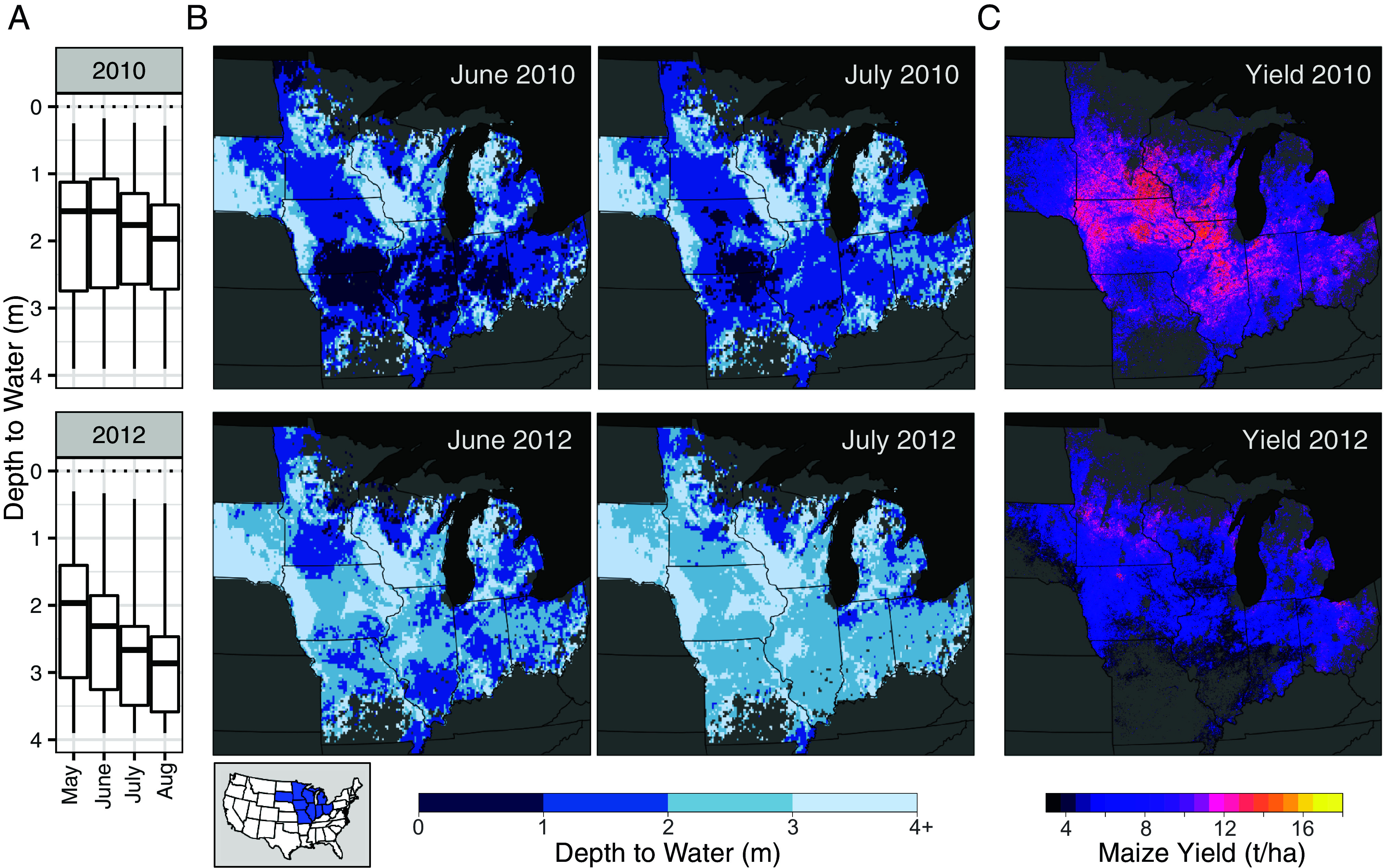
Study area and example groundwater and crop yield data. (*A*) Region-wide distribution of groundwater levels by month during the growing season, based on the 1D Enhanced Crop Model. (*B*) Simulated groundwater levels in June and July. (*C*) Annual yield maps derived from satellite data. *Top* row: 2010, the wettest year in our study. *Bottom* row: 2012, the driest year in our study. The *Inset* map (*Bottom* *Left*) shows the nine study states (blue) within the continental United States.

Here, we provide field-scale empirical evidence for shallow groundwater effects on maize yields across the US Corn Belt. We used validated satellite yield observations at subfield (30 m) resolution generated via the Scalable Crop Yield Mapper (SCYM) spanning a nine-state study region over 20 y (1999 to 2018). We combined these with best-available estimates of dynamic, monthly water table depths from an Enhanced Crop Model (Fig. 1), which improves upon the APSIM crop model to better simulate shallow water tables and root growth responses. We then tested for groundwater yield subsidies and penalties in three ways: 1) a comparison of observed yields against simulated yields from the Enhanced Crop Model and those from a standard implementation of APSIM (hereafter, the Standard Crop Model); 2) an inferential machine learning framework to isolate the yield impacts as a function of groundwater levels based on observational data, accounting for variable weather and soil properties; and 3) an analysis of yield stability. Within our inferential framework, we also leverage our paired crop simulations to assess the relative importance of two competing underlying mechanisms, direct root water uptake and enhanced soil moisture through capillary rise. Based on thresholds identified in our inferential model, we then quantified where and how often crop water stress conditions aligned with groundwater levels to create conditions for groundwater yield impacts, as well as their estimated regional monetary impact.

## 1. Results and Discussion

### 1.1. Crop Simulations with Groundwater Better Matched Observed Yields.

**Fig. 2.**
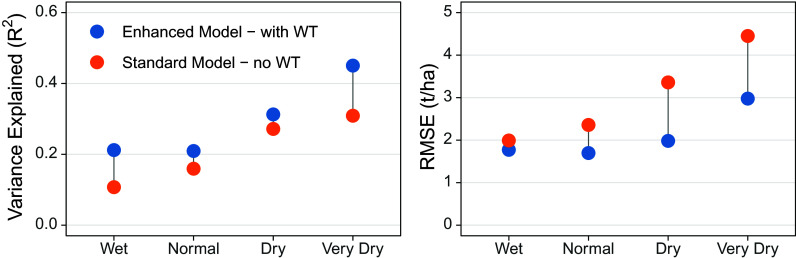
Agreement between satellite yield observations and crop simulations by weather class. The Standard Crop Model refers to a traditional implementation of the APSIM crop model; the Enhanced Crop Model refers to our improved model that accounts for interactions with the water table (WT). Weather classes were defined based on the July water deficit (precipitation minus PET) for each simulation grid-year. Wet = positive values where precipitation exceeded PET; Normal = water deficit was within −50 and 0 mm; Dry = deficits between −150 and −50 mm; Very Dry = deficits > −150 mm. RMSE = root mean squared error.

We found that the Enhanced Crop Model with water table improvements better matched observed satellite-derived yields than the Standard Crop Model (Fig. 2). Overall, the Enhanced Crop Model explained more variation in observed yields (R^2^ = 0.39 vs. 0.36) and reduced the RMSE by 37% from 3.1 to 1.9 t/ha compared to the Standard model. We found the largest improvements in R^2^ in the wet and very dry weather classes, with R^2^ doubling from 0.10 to 0.21 in wet conditions and increasing by 50% in very dry conditions from 0.30 to 0.45. The largest decreases in RMSE occurred in dry and very dry weather conditions, with reductions of 42% and 33% percent, respectively (Fig. 2). This indicates that groundwater access influences maize yields in the US Corn Belt, and that crop simulations can perform more realistically if they are designed to better capture shallow groundwater dynamics, particularly in years with hydrological extremes. In addition to yields, properly accounting for groundwater can improve accounting of nitrogen loss, soil carbon assessments, and connections with land surface models (11, 18).

### 1.2. Yield Response to Groundwater Levels.

#### 1.2.1. Mid-late season (July) water table as key time period.

Our machine learning models of maize yields using random forests performed well, explaining 69% of out-of-bag yield variance for SCYM pixel-based yield observations (mean of squared residuals = 1.9 t/ha) and 91% of yield simulations from the Enhanced Crop Model (0.55 t/ha). Variable selection identified July groundwater levels as the most relevant groundwater period for yield response; no other month or the annual time step was selected. Generally, drought impacts to maize are most often observed in July, which coincides with crop growth at or near pollination (3, 36). Other studies have found impacts from excess moisture occur most often in June due to the combination of crop growth in the early vegetative stage and typically higher rainfall totals, although July follows closely behind and is also susceptible to excess moisture (7). July thus captured impacts from both excess water and water stress, and the inclusion of additional months (e.g., June) did not substantially improve model performance.

#### 1.2.2. Groundwater yield subsidy zones.

**Fig. 3.**
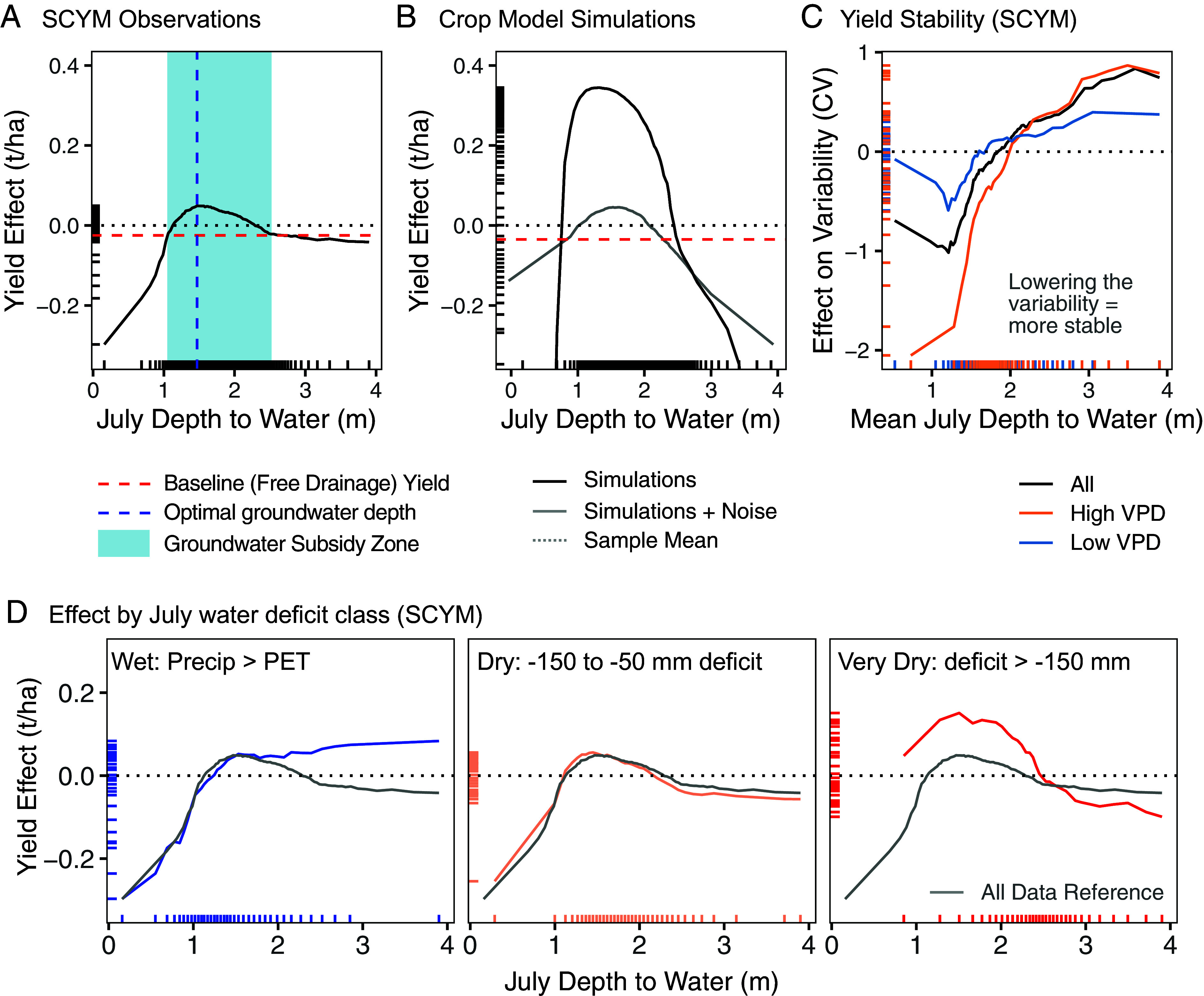
Maize yield response to changes in groundwater levels based on ALE plots from random forest models. (*A*) Observational random forest model using SCYM satellite yields. The blue shaded region denotes the subsidy zone, where yields are above baseline free drainage yields (red dashed line). (*B*) Simulation random forest model using the Enhanced Crop Model. Black line = model simulations. Gray line = simulation with introduced noise approximating the agreement between the Enhanced Model water table estimates and validation data (r = 0.6). (*C*) Yield stability by groundwater levels based on SCYM observations. Variability was measured as the coefficient of variation (CV; unitless) at each of 135,000 sample locations between 1999 and 2018 when maize was present. VPD = vapor pressure deficit; high (low) indicates the *Top* (*Bottom*) tercile of VPD based on the 1999 to 2018 mean for each location. (*D*) Yield effect by July water deficit (precipitation - potential evapotranspiration) groupings. The gray line reproduces the curve for all samples displayed in panel (*A*). For all subplots, the effect is presented relative to the population mean of the variable in question (e.g., “mean yield,” dotted line at 0), a convention of ALE plots. Tick marks along the axes represent the data distribution along each respective axis.

When the SCYM yield response to July depth-to-groundwater is visualized with the accumulated local effects (ALE) plot, we found a nonlinear response with optimum yields at 1.5 m and yield declines on both sides of this optimum (Fig. 3*A*). The ALE plot also displayed a lower threshold at 2.5 m where yields became invariant to groundwater depths. This represents “free drainage” conditions where groundwater is too deep to meaningfully contribute supplemental water to the root zone, and we assigned this plateau as the baseline free drainage yield (19). The yield effect remained above this baseline until water levels became shallower than 1.1 m, indicating that the groundwater subsidy zone for maize in this system is between 1.1 and 2.5 m (Fig. 3*A*). When groundwater levels are shallower than this zone, we found a rapid yield decline, indicating a groundwater penalty from excess water. The nonlinear response of yield to groundwater depth is also evident if we use USDA county-level reported yields (and county-averaged covariables) rather than field-scale SCYM estimates (*SI Appendix*, Fig. S1*A*).

Our findings are consistent with biological understanding of maize root distributions and previous work. Previous work has identified optimum groundwater depths for maize between 1.1 and 2.0 m in simulations of southern Wisconsin (26), between 1.40 and 2.45 m from observational data in Argentina (37), and little interaction below 2.5 m in semiarid and arid regions (23, 38).

#### 1.2.3. Magnitude of yield impacts.

While SCYM satellite observations were able to delineate characteristic zones for yield subsidies and penalties, the magnitude of these effects is less clear. Compared to free drainage conditions, the maximum yield subsidy estimated with SCYM yields was approximately 0.074 t/ha (bootstrapped 95% CI: [0.071 t/ha, 0.078 t/ha]; *SI Appendix*, Fig. S1*B*), or 0.7% of the mean yield in the study sample (10.8 t/ha). This response is considerably smaller than that seen for simulated yields from the Enhanced Crop Model, where we estimate a 0.37 t/ha (3.4%) yield subsidy based on the same free drainage baseline as in the SCYM model (Fig. 3*B*). We use the SCYM-defined baseline since the characteristic flattening of the yield effect indicative of free drainage (19) is not clearly defined in the modeled results, possibly due to stochasticity arising from the low density of samples at deeper water table depths or model simulation shortcomings. The clear transition to free drainage conditions in the SCYM observations matching theoretical expectations as outlined by Zipper et al. (19) underscores the need to empirically evaluate the subsidy phenomena, as we do here.

The discrepancy in effect size magnitude among the two methods may be due to attenuation. While groundwater levels are perfectly known for the simulated yields (since the yields and groundwater levels are taken from the same simulation), SCYM yields can only be matched to an imperfect measure of true 30 m resolution groundwater levels; the Enhanced Crop Model groundwater levels are at 7.5 km resolution and are a 1-D simulation unable to account for lateral flow in the subsurface. As noted in Section 3.2, the correlation between field-measured groundwater and simulated values was 0.62. This measurement error can result in substantial dilution of the estimated yield response, a phenomenon known as regression dilution (39). To explore this possibility, we repeated the Enhanced Crop Model analysis but replaced groundwater levels with a “noisy” groundwater measure that had a correlation with the simulated values equal to the estimated accuracy of the Enhanced Crop Model (r = 0.6). We found that introduced noise diminished the effect, resulting in a yield subsidy similar to the observational data (Fig. 3*B*).

As a result of this comparison, we conclude that the subsidy estimates derived from both methods are relatively consistent, and we hypothesize that the simulation-derived magnitude of 3.4% is likely closer to the true (average) value. This would be similar to the suggested 6% average groundwater contribution from Rizzo et al. in this region (25). On the higher end, an observational study in Argentina found maize yields tripled when groundwater was accessible, likely because of the drier climate and high rainfall variability (37).

When we break results down by July water deficit classes, we also find differential groundwater yield impacts. We found that the yield subsidy approximately doubled for “very dry” conditions relative to the full sample (0.22 t/ha vs. 0.1 t/ha; Fig. 3*D*). This magnitude is still well below the 24% yield increase found in drought conditions by Rizzo et al. (25), but their approach used a crop simulation with unlimited water access as a proxy for accessible groundwater; they then compared yields obtained under this “unlimited water” simulation with a traditional simulation constrained by rainfall data, attributing the yield differential to groundwater. While a good early approximation, this approach likely overestimated the availability of groundwater, particularly during dry conditions which would concurrently lower the water table. Ultimately, the estimated magnitude of groundwater subsidies depends on the amount of water stress present during the study period as much as the presence of groundwater (7).

In contrast to this weather influence on the groundwater subsidy, we found that wet July conditions did not worsen yield penalties from very shallow groundwater within the penalty zone (Fig. 3*D*), likely because the root zone was already saturated. This corroborates results finding that areas prone to water excess see yield losses across a range of seasonal rainfall, including normal and even drier than average conditions (13). While yields at very shallow water table depths demonstrated the largest yield impacts (Fig. 3), the mean depth to water for observations in our sample located within the penalty zone was 0.85 m, corresponding to more minor yield decrease of ~1%.

#### 1.2.4. Yield stability due to groundwater presence.

Overall, the mean coefficient of variation (CV) of our SCYM study sample was 18.1% (interquartile range: 13.7 to 21.4%). Compared to locations with deeper groundwater, yield stability increased where shallow groundwater was present, as demonstrated by a decrease in CV at shallower water table depths through the upper limit of the subsidy zone (~1 m, Fig. 3*C*). Note that Fig. 3*C* depicts mean July groundwater depths during the 20-y study period; therefore, locations with a mean depth of 3 m, for example, likely still see water tables in the subsidy zone in some years (but not all, thus increasing the yield variation at these locations). The stabilizing effect of groundwater availability was stronger at locations with mean vapor pressure deficit in the highest tercile of our dataset (a 2.8% decrease in CV between 3.5 and 1 m depths), and weakest at locations with mean vapor pressure deficit in the lowest tercile of our dataset (~1% decrease; Fig. 3*C*). This further demonstrates that shallow groundwater reduces heat-induced water stress and thus increased yield stability in locations with groundwater access. Rizzo et al. (25) similarly found lower CVs in their model scenarios that included unlimited water (9 vs. 14%).

#### 1.2.5. Estimating the relative contribution of underlying mechanisms.

As mentioned above, shallow groundwater within or near the root zone can supplement plant water use in two primary ways: direct root water uptake and/or enhanced soil moisture through capillary rise from the water table. Generally, the capillary fringe can influence soil moisture up to 1 m above the water table (40). Because the Standard Crop Model simulates only the top 2 m of the soil profile and considers water that percolates below 2 m as water lost to free drainage, the Standard Model’s estimates of soil moisture are driven by “top–down” influences on soil moisture such as rainfall and evapotranspiration. The Enhanced Crop Model, on the other hand, captures both “top–down” and “bottom–up” sources of soil moisture because it extends the bottom boundary to 4 m and includes a specified hydraulic head as the bottom boundary condition (Section 3.2). This difference allows us to estimate the relative contribution of direct and indirect mechanisms to groundwater yield subsidies. First, by comparing between the models, we found that water table contributions increased monthly soil moisture in the top 1 m by a median value of 30.8% across all grid-month-years, with an interquartile range of 8.9 to 66.8%. This is comparable to a 21% increase estimated by a soil hydrological model parameterized for the Nebraska Sand Hills (41).

For our primary model of groundwater yield effects presented above (e.g., Fig. 3), we aimed to estimate the total groundwater contribution to yields from both direct and indirect mechanisms. We therefore used soil moisture estimated from the Standard Crop Model as a covariable to account for soil moisture from “top–down” sources only, as soil moisture is a primary driver of crop performance (14). To isolate the relative contribution of each mechanism, we then ran the model again, instead using soil moisture from the Enhanced Crop Model. We found that the yield effect at the optimum water table depth is reduced by 45% in this model. From this, we estimate that, in this system, just over half of the total groundwater contribution is due to direct root uptake (~55%), while just under half is due to enhanced soil moisture through capillary rise (~45%).

### 1.3. Prevalence of Groundwater Yield Impacts.

**Fig. 4.**
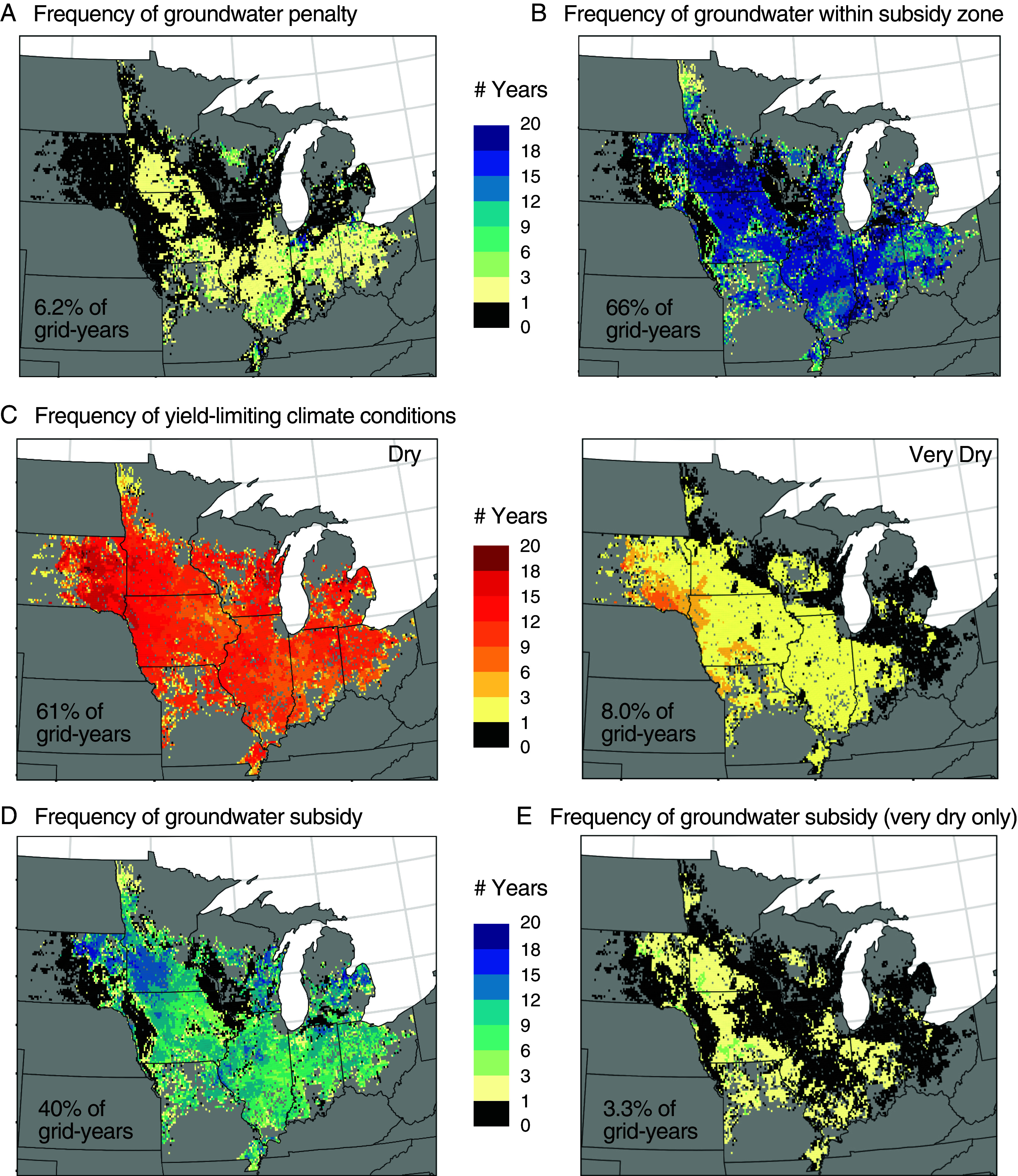
Prevalence of groundwater yield impacts by climate conditions based on the Enhanced Crop Model gridded simulations for 1999 to 2018. (*A*) Number of years by model grid cell with groundwater penalty conditions, defined by our models as July groundwater levels <1.05 m. (*B*) Number of years with groundwater levels within the identified subsidy zone. (*C*) Number of years with July water deficit (precipitation minus PET) below −50 mm (“Dry,” *Left*) and −150 mm (“Very Dry,” *Right*). (*D*) Number of years with groundwater subsidy conditions, when groundwater within the subsidy zone coincides with yield limiting climate conditions. (*E*) Number of years with groundwater subsidies during Very Dry conditions.

We found July groundwater levels within the penalty zone (<1.1 m) in 6.2% of grid-years in the Enhanced Crop Model (Fig. 4*A*). Within our study period of 1999 to 2018, groundwater yield penalties occurred more frequently in southern Illinois, northwest Indiana, northwest Ohio, and southeast Missouri. Still, penalty conditions appear to be limited, likely due to extensive tile drainage across the region. It’s worth noting, however, that total agricultural production may be more sensitive to wet extremes than our analysis detects since excess water during the planting period can decrease total area planted.

We found July groundwater levels within the identified subsidy zone of 1.1 to 2.5 m in 66% of model grid-years (Fig. 4*B*). While this indicates that groundwater is frequently accessible, a groundwater subsidy only occurs when access to groundwater alleviates a yield-limiting water deficit. We found that yields decline from the optimum when July water deficit falls below −50 mm (*SI Appendix*, Fig. S2). In our sample, 61% of grid-years met this climate condition, while 8.0% met the more stringent threshold of −150 mm in our “very dry” classification (Fig. 4*C*). When considering both prerequisites for groundwater yield subsidies—concurrent groundwater access and climate stress—we found conditions suitable for groundwater yield subsidies in 40% of model grid-years, and only 3.3% of grid-years for “very dry” weather conditions. This indicates groundwater levels are not always sufficiently high to provide supplemental water when needed, particularly as they often lower in dry years (e.g., Fig. 1). Across all grid cells, the mean frequency of subsidy years was 34% (6.77 y of 20); grid cells within Minnesota had the highest mean frequency of subsidies (42%) followed closely by Illinois (40%), while South Dakota had the lowest (23%; Fig. 4*C* and *SI Appendix*, Fig. S3). Seventy-five percent of grid cells received a subsidy in at least 10% of study years, and the annual geographic extent varied between a minimum of 17% and a maximum of 66% of grid cells.

Based on grid-specific annual maize production from SCYM, inflation-adjusted maize prices (42), our designations of penalty, subsidy, and strong subsidy (e.g., “very dry”) conditions, and yield effect magnitudes from the inferential model (−1%, 3.4%, and 7.8%, respectively), we roughly estimate the overall monetary impact of groundwater-yield interactions to be approximately $10 billion during our study period. This includes $10.4 billion in total subsidies and $453 million in total penalties, although we note this likely underestimates the full cost of yield penalties since our analysis does not quantify production declines due to prevented planting or early season groundwater levels that are not in our statistical model. The monetary value from groundwater subsidies ranged from a low of $164 million in 2016 to a high of $1.2 billion in 2011, largely due to price variability. *SI Appendix*, Fig. S4 provides a time series of annual maize production, prices, and estimates of monetary impacts.

### 1.4. Climate Change and Sustainability Implications.

Climate change in the region is expected to bring increased variability in precipitation patterns that are likely to exacerbate groundwater penalties through excess rainfall (43, 44) as well as create conditions for groundwater yield subsidies through mid-season water stress (45). Groundwater could partially buffer for water and/or heat stress in the region. However, we cannot assume that water tables will be within the subsidy zone if overall climate trends hotter and drier; for example, in the Hungarian Plains, groundwater yield subsidies for maize have decreased over the past 50 y as groundwater levels fell due to land use and climate change, reducing yields as much as 11.6% (29). To date, consideration of groundwater impacts to yields has been largely lacking in studies of climate impacts to crop production in the US Corn Belt. The importance of considering groundwater impacts extends beyond crop production because water is the main driver of soil nitrogen and carbon cycling and therefore sustainability assessments (46, 47). While a more precise understanding is still needed to properly quantify groundwater’s role, there is sufficient evidence to suggest that changes in climate will likely be mediated through groundwater presence and overall soil water hydrology.

## 2. Conclusions

Our study contributes to a growing body of research supporting consequential contributions of groundwater to grain yield outcomes in the US Corn Belt. Given these contributions, it may be important for climate adaptation efforts to consider where crops have more limited access to groundwater and thus have higher sensitivity to extreme heat. It will also be important to consider how climate change will modify the depth of groundwater throughout the year, thus potentially exacerbating or reducing anticipated climate effects based on historical data. Understanding optimal groundwater depths may also inform the potential adaptation of tile drain systems to support subirrigation delivery during periods of water stress (48). Beyond yield impacts, an improved understanding of shallow water table dynamics can help with broader landscape management. For example, nitrogen loss is higher with shallower water tables, increasing both the economic optimum nitrogen fertilization rate as well as nutrient export to aquatic systems (11).

While we build upon previous work and provide field-level observational evidence for groundwater yield impacts across the region, our work does have several limitations that highlight areas for future study. First, the design of our study is likely better suited to estimate groundwater yield subsidies than penalties. Our variable selection process selected only July water tables, but an analysis focused solely on groundwater penalties would likely benefit from an examination of water tables before and during the planting season, or April to June (49). Similarly, high water tables can impact maize production in this region by preventing planting altogether (9), which our study does not capture. The widespread use of tile drains in the study region further complicates the interpretation of groundwater yield impacts and raises a suite of interesting questions: How much do tile drains already mediate groundwater penalties in the region, and where might new tile installation be most advantageous to reduce penalties? What tile depth and spacing are most effective, and could tile design improvements optimize groundwater-yield effects? Do current tile drains reduce the potential for groundwater yield subsidies by lowering the water table? One might address some of these questions by running crop simulations with and without tile drains, but anecdotally, in our experience this often creates difficulty with water balance convergence in the model, suggesting the presence of tile drains may be necessary to successfully grow maize in many parts of this region. This is further supported by the large investments in tile drainage over the past century and ongoing expansion (50, 51). Another key knowledge gap is the locations and density of tile drains themselves. Although the USDA provides county-level estimates of drained area, and there are efforts that distribute this spatially based on soil and topography rules (52), actual locations remain unknown for the full region. There is promising early work using remote sensing to map tile drains for small regions (50); scaling this or similar work across the Corn Belt would help improve our understanding of drainage effects.

Our work further identifies a need for increased spatial resolution in groundwater depth data to better quantify the magnitude of groundwater yield impacts. We hypothesize that the estimated magnitude from our high-resolution (30 m) observational yield data is attenuated due to the lower resolution of our modeled groundwater levels (7.5 km), leading to averaging effects. This spatial mismatch could be reduced either through an improved, high-density observational network or with more spatially refined coupled surface-groundwater landscape hydrology models specifically designed to capture tile-dominated agricultural systems (e.g., ref. 53). The latter could improve upon our 1D simulation by including lateral flows, but these are currently complex and computationally expensive to run at high resolution for large areas. Ultimately, to better quantify groundwater yield impacts, we still need better information on groundwater presence and spatiotemporal dynamics.

## 3. Methods

### 3.1. Study Area.

The US Corn Belt is characterized by commercial-scale agriculture dominated by maize-soybean rotations, producing about 30% of global maize and soybeans. Here, we focus on nine states with largely rainfed agriculture (Fig. 1). The regional climate is predominantly humid continental (54), receiving the majority of annual rainfall during the growing season. Although crop water needs are typically met without supplemental irrigation, the region experiences occasional drought, particularly during the mid-to-late growing season, and high temperatures that inhibit crop yields (5). Although still rare on the landscape, irrigation is used in some parts, particularly in sandy soils or for high-value crops (48). In recent years, irrigation has seen some expansion (42). Here, we considered only rainfed fields based on remotely sensed irrigation maps (Section 3.3).

Shallow groundwater underlies much of the region. Groundwater maps generated via steady-state hydrological models, which estimate the water table using long-run climate averages and largely ignore human land use, show extensive regions with groundwater within the top 1 m (15, 31). In practice, however, the hydrology has been substantially altered through drainage tiles placed ~0.8 to 1.2 m below the surface (32, 55). Drained area has undergone significant expansion since the late 1990s (50, 52) now covering as much as 60 to 90% of county cropland across central Corn Belt counties (42) and approximately 50% of production fields in the region overall. Groundwater levels are dynamic, responding to patterns in rainfall and atmospheric water demand (Fig. 1). Maize typical rooting depths range from ~90 to 150 cm and are positively correlated with water table depth (33, 34).

### 3.2. The Enhanced Crop-Hydrology Model.

Crop simulation models traditionally have difficulty accurately representing crop growth and yield responses to subsurface water. They often fail to capture nonlinear yield responses to precipitation, with a noticeable absence of yield penalties in waterlogged conditions (7). Moreover, standard modeling practice is to simulate the top ~2 m of the soil profile and consider water that percolates below this as drainage out of the model, thus failing to account for additional water stored in the deep soil, which may become available to plant roots through capillary action from the water table.

Here, we coupled a previously calibrated and validated APSIM crop model (55–57) with pSIMS software (58) to perform regional-scale simulations of shallow water table. This coupled APSIM-pSIMS modeling system (hereafter referred to as the Enhanced Crop Model) better accounts for subsurface hydrology in three core ways: 1) it uses a maize crop model (as opposed to a generic vegetation model with unlimited water uptake) and accounts for root growth inhibition and waterlogging impacts on grain yield under shallow water table conditions (11, 59); 2) it extends the simulation to 4 m below the soil surface to better represent water table depth fluctuations, especially in dry conditions (55), with a specified hydraulic head based on gSSURGO water table depth (60) as the bottom boundary condition; and 3) it simulates subsurface tile drainage when present. Thus the Enhanced Crop Model functions as a 1D crop-hydrology model with a daily time step, simulating crop growth and yield with a better representation of vegetation–soil–groundwater interactions and land use modifications than a standard APSIM model.

Here, we discretized the study region into 0.0833 degree (~7.5 km) regular grid cells, and simulations were run for each cell with agricultural presence (16,179 active model cells). Soil properties were taken from the dominant conditions in each cell (gSSURGO). Tile drainage status was set based on the AgTile dataset (52), which spatially distributes 2017 county-level tile drained area based on topography and soil drainage classes. Historic daily weather data were acquired from Environmental Mesonet (Reanalysis product). For each simulation, we tracked annual crop yield and monthly groundwater levels. Simulated groundwater levels thus vary in space and time, with regionally high water tables in wet years and low water tables in dry years; typically, groundwater levels are highest near planting and lower during the growing season (Fig. 1 and *SI Appendix*, Figs. S5 and S6). The Enhanced Crop Model captures historical grain yields with a RMSE of 1.2 t/ha (*SI Appendix*, Fig. S7) while having sensible water table fluctuations across space and time (*SI Appendix*, Figs. S5 and S6). When compared to 6,232 ground truth water table observations from 37 sites, the Enhanced model showed reasonable agreement with monthly groundwater levels (r = 0.62, RMSE = 0.80 m).

We also ran an equivalent calibrated APSIM-pSIMS model lacking these enhancements (hereafter referred to as the Standard Crop Model) to simulate yields without accounting for groundwater. Subsequent analysis was limited to model cells containing at least 10 percent annual maize area as indicated on the USDA’s annual Cropland Data Layer (CDL) (61), resulting in a total of 226,394 grid-year observations between 1999 and 2018 (range: 9506 to 10,780 grids cells per year).

### 3.3. Yield Data.

We obtained maize yield observations from a previously published spatial dataset providing yields across the study region annually for 1999 to 2018 at 30 m resolution (62). This dataset was developed using the SCYM approach (63), which estimates crop yields based on satellite data and region-specific crop model simulations. When validated against an extensive ground dataset spanning over 375,000 maize fields between 2008 and 2018, SCYM captured 40% of yield variation at the pixel level and 69% when aggregated to the county scale (62). Notably, SCYM was able to reproduce linear and nonlinear relationships between crop yields and field attributes not included in the SCYM model, including management practices such as sowing date and density in addition to physical properties such as soil quality and soil water holding capacity (62).

We created two SCYM yield datasets for use in this study. For the first, we extracted annual SCYM yields averaged over the crop model grid for direct comparison with the crop model simulations. For the second, we generated a dense point sample at the 30-m pixel-scale across the study region, randomly sampling 135,000 locations which grew maize at least 10 times between 1999 and 2018. Prior to sampling, we first excluded irrigated locations with the LANID dataset, which provides annual irrigation status for the continental United States based on Landsat data (64).

### 3.4. Analyses.

#### 3.4.1. Observational evaluation of the Enhanced Crop Model.

We used the grid-averaged SCYM yields to evaluate simulated yields from both the Standard and Enhanced Crop Models with this observational yield data aggregated to subcounty, 7.5 km resolution. If the enhancements in subsurface hydrology within the Enhanced Crop Model produced more realistic yield estimates, we would see a stronger agreement between this model and SCYM satellite yield data than for the Standard Crop Model. We quantified agreement between the datasets using the squared correlation (R^2^) and root mean square error (RMSE) for all grid-years. We compared both overall agreement and agreement for climate subsets based on the July water deficit, which is defined as monthly precipitation minus potential evapotranspiration (PET) (and thus, negative values indicate a deficit) and can be considered a nonstandardized version of the Standardized Precipitation Evapotranspiration Index (43, 65). July is the most sensitive period for maize growth, and water deficit is strongly correlated with yield impacts from water stress and a key driver in soil moisture variability in the study area (3, 7, 25, 36, 66). We calculated July water deficit for each year using nominal 4 km resolution GRIDMET climate data (67). We then created four classes based on July water deficit: “wet,” defined as positive values where precipitation exceeded PET; “normal” where the water deficit was within −50 and 0 mm; “dry” with deficits between −150 and −50 mm; and “very dry,” with deficits >−150 mm.

#### 3.4.2. Inferential machine learning.

To isolate the impact of groundwater levels on crop yields, we developed random forest models (68) to predict yields using groundwater levels generated from the Enhanced Crop Model and a suite of environmental covariates describing weather and soil conditions. Random forests are an ensemble machine learning approach which use consensus predictions from many regression trees each trained on a subset of data and predictor variables. They are therefore nonparametric, robust to multiple colinear variables, able to capture high-order interactions, less prone to overfitting, and relatively straightforward to implement (68, 69). Random forests excel at prediction tasks, consistently outperforming multiple linear regression in climate and agriculture applications, including maize yield prediction (70, 71).

ALE plots (72) are a recent method used to summarize random forest model response to individual variables. ALE plots discretize the data along the predictor variable and estimate the local effect of increases in that variable. These local effects are then combined or “accumulated” into the ALE plot to visualize the global effect, which are centered at the mean model prediction (72). By focusing on local effects, ALE avoids interpretability problems of the more common partial dependence plots (73), which can be misleading when predictors are correlated. Recent work has used ALE plots to examine weather effects on US maize yields (74).

To compare groundwater yield effects identified via simulations and observed yields, we developed separate random forest models for the Enhanced Crop Model and for SCYM yield maps. For SCYM, we used the pixel-level yield sample to avoid averaging effects. The full dataset of 135,000 locations sampled in all maize years (Section 3.3) resulted in 2,063,340 pixel-year observations. To reduce data size for this analysis, we generated a random subsample of 35,000 observations per year, resulting in 700,000 pixel-year yield observations for this analysis.

We then assembled 37 candidate predictor variables, including 29 weather, soil, and topography variables (*SI Appendix*, Table S1), monthly Enhanced Model water table levels for May to August each year, and annual Enhanced Model water tables. For soil moisture in the top 1 m, we used monthly estimates from the Standard Model, which is driven by “top–down” sources of soil moisture such as rainfall and evapotranspiration and does not account for groundwater contributions to soil moisture through capillary rise. Because we aimed to quantify total yield impacts from groundwater, this lack of groundwater-sourced soil moisture make it ideal for our analysis. To improve computational efficiency, we first performed variable selection using a multivariate adaptive regression spline model (75) with degree = 2 as implemented in the “earth” R package (76). The final selected set of 19 variables and their data sources are listed in *SI Appendix*, Table S2. The random forest model was then implemented with the “randomForests” R package (77) with 100 trees, six variables per split, and nodesize = 10 to avoid overfitting. Model performance was evaluated on out-of-bag samples. We then visualized the effect of individual predictors using the ALEPlot R package (78). For the Enhanced Crop Model, we used the same 19 variables identified for the SCYM analysis but used simulated yield output and grid-averaged values of the 19 covariables.

#### 3.4.3. Stability analysis.

Shallow groundwater may decrease interannual yield variability, particularly in locations where it provides a groundwater subsidy (25, 29). To examine the relationship between yield stability and groundwater levels, we first calculated the CV for each of the 135,000 pixel-level sample locations throughout the region, defined as the SD/mean * 100. We then trained a random forests model following the method described in Section 3.4.2 and used an ALE plot to examine the relationship between yield variability and mean groundwater levels for each sample location from the Enhanced Crop Model.

#### 3.4.4. Prevalence of groundwater yield impacts.

Opportunities for groundwater yield subsidies arise when yield-limiting climate water deficits occur in the presence of accessible groundwater. To quantify the spatiotemporal frequency of these conditions in our study, we first identified thresholds for groundwater subsidies and water stress based on ALE plots for the SCYM random forests model. We report on these thresholds in Section 1.3. We then identified and tallied Enhanced Crop Model grid cells which met these conditions to create regional frequency maps of groundwater subsidies. For groundwater penalties, we used groundwater thresholds only.

## Supplementary Material

Appendix 01 (PDF)

## Data Availability

Data supporting the analyses and R code for theanalyses and figure generation are available on Zenodo at https://doi.org/10.5281/zenodo.11393498 (79).
