# Supplementary material for: Observational evidence for groundwater influence on crop yields in the United States
Source: Proc Natl Acad Sci U S A. 2024 Aug 26;121(36):e2400085121. doi: 10.1073/pnas.2400085121 (PMC11388334; doi:10.1073/pnas.2400085121)
Supplement: Supplementary file 1 — Appendix 01 (PDF) [file pnas.2400085121.sapp.pdf]

PNAS

Supporting Information for
Observational evidence for groundwater influence on crop yields in the United States.

Jillian M. Deines, Sotirios V. Archontoulis, Isaiah Huber, David B. Lobell

Jillian M. Deines
Email: jill.deines@pnnl.gov

This PDF file includes:

Figures S1 to S7
Tables S1 to S2
SI References

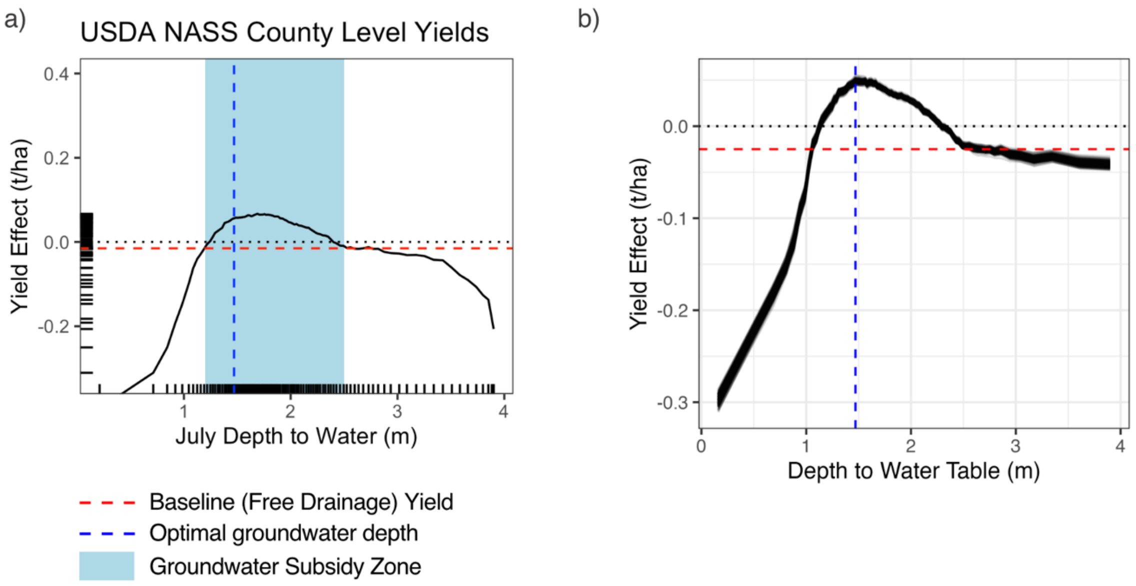

Fig. S1. Maize yield response to change in groundwater levels from a) county-level yield statistics and b) bootstrapped SCYM pixel-level data. (a) Subsidy relationship derived from county-level yield statistics. This figure mirrors Figure 3a, but instead of SCYM sub-field yield observations, it uses county-level yield statistics available from USDA NASS and county-averaged covariables obtained by averaging over all SCYM samples within each county. The response curve is based on an accumulated local effects (ALE) plot from a random forest model fit to the county data. Here, we retain the same groundwater subsidy zone, baseline yield, and optimal groundwater depth marker locations (shading and dotted lines) as in Figure 3a to facilitate comparison. Overall, we do see a slightly deeper optimum depth (1.7m) and subsidy zone (1.2-2.5) for the county data than for SCYM observations. (b) Estimates of the yield – groundwater level relationship were relatively consistent across 200 bootstrappings of the ALE plot in Figure 3a.

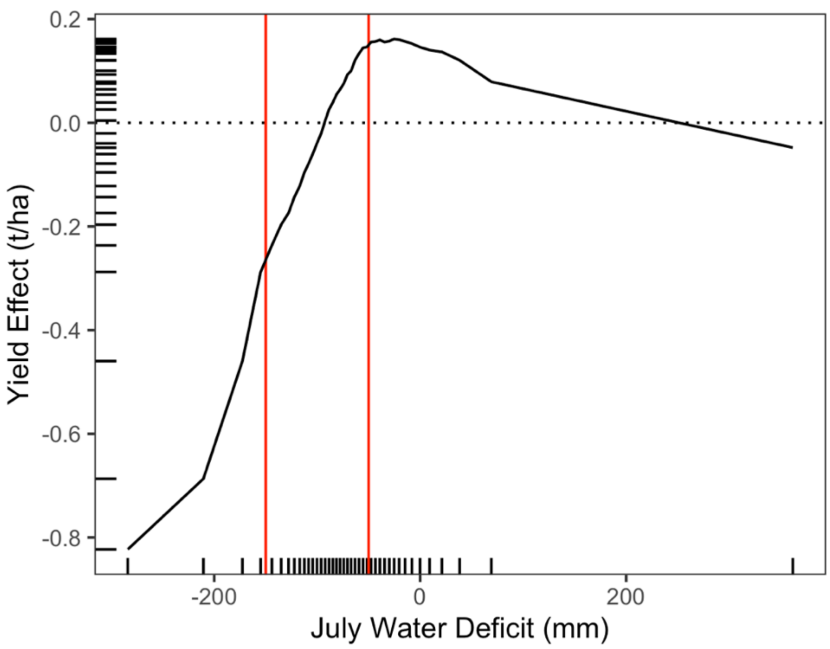

Fig. S2. Yield response to July Water Deficit based on accumulated local effects (ALE) plots from the observational random forest model. Water deficit is defined as precipitation minus PET. Dotted line indicates the sample mean yield. Red lines indicate thresholds of -50 mm and -150 mm.

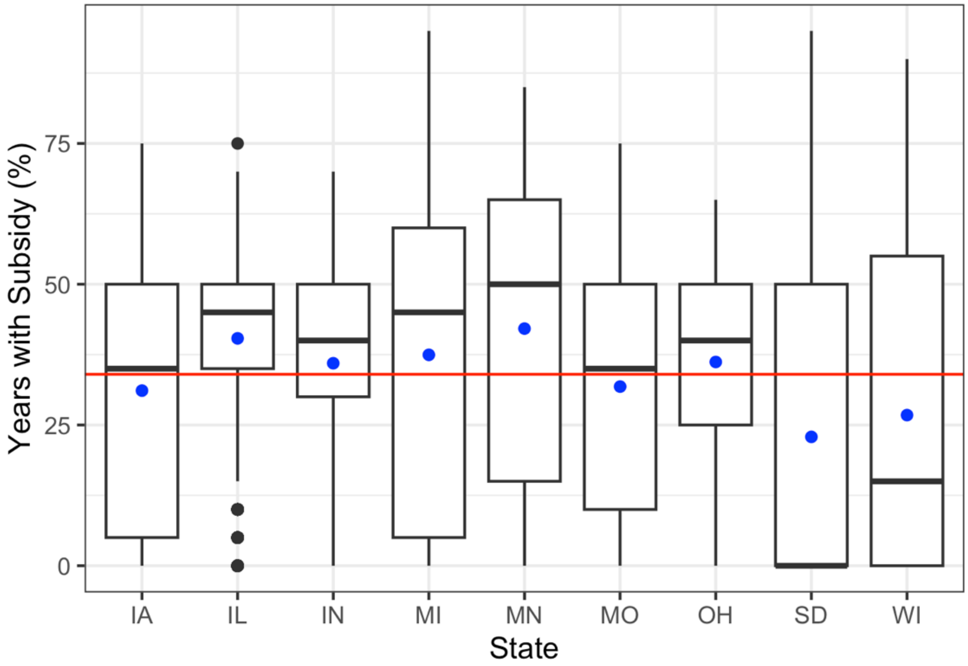

Fig. S3. Distribution of groundwater subsidy frequency by model grid cells within each state. Blue dot shows the state mean, while the red line shows the overall mean (34%) for the study area.

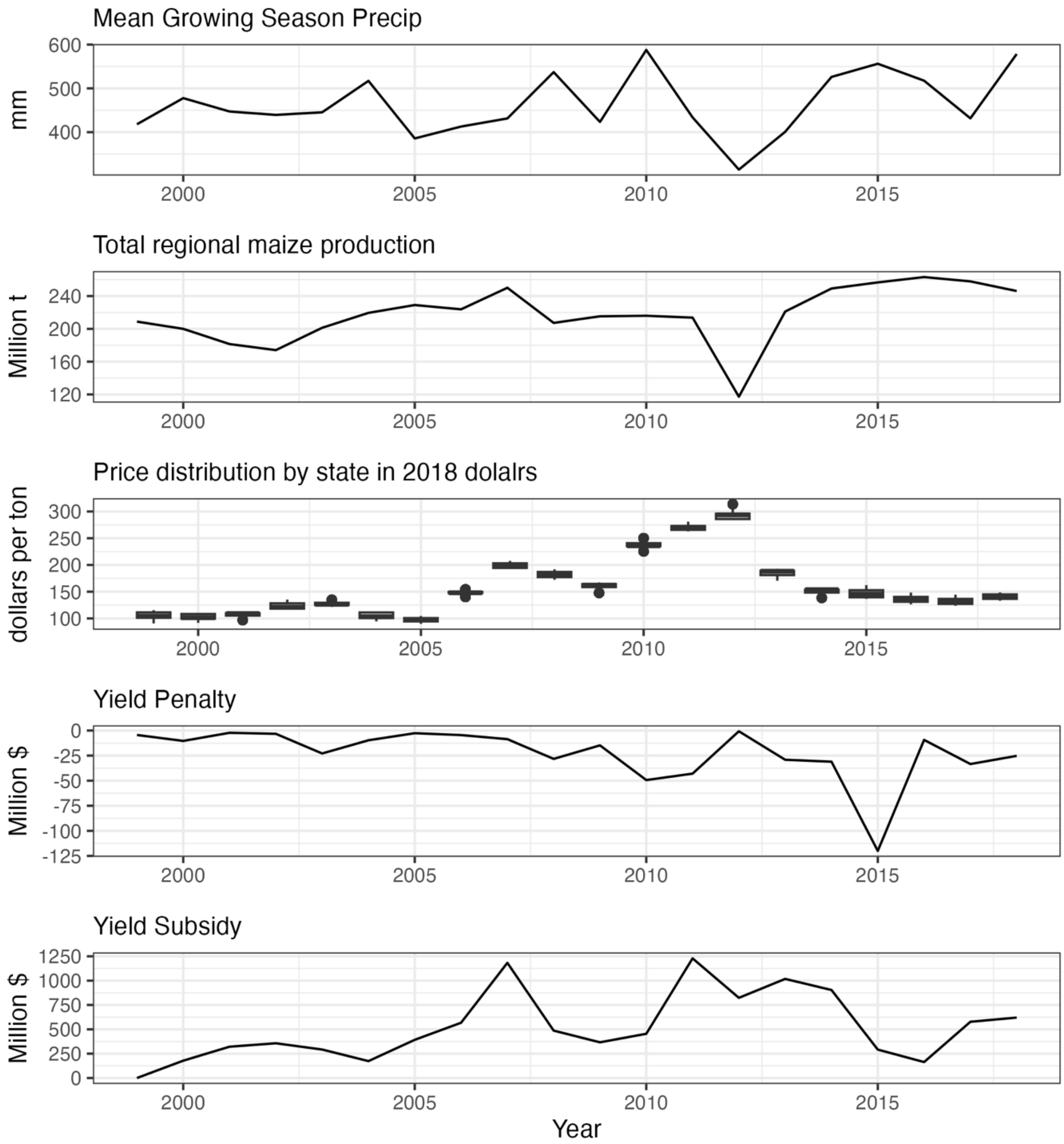

Fig. S4. Time series of the estimated monetary impacts of groundwater interactions. Annual state-specific price data comes from USDA National Agricultural Statistics Service. Annual maize production is derived from SCYM yields and area. Growing season precipitation is the sum of precipitation in June, July, and August.

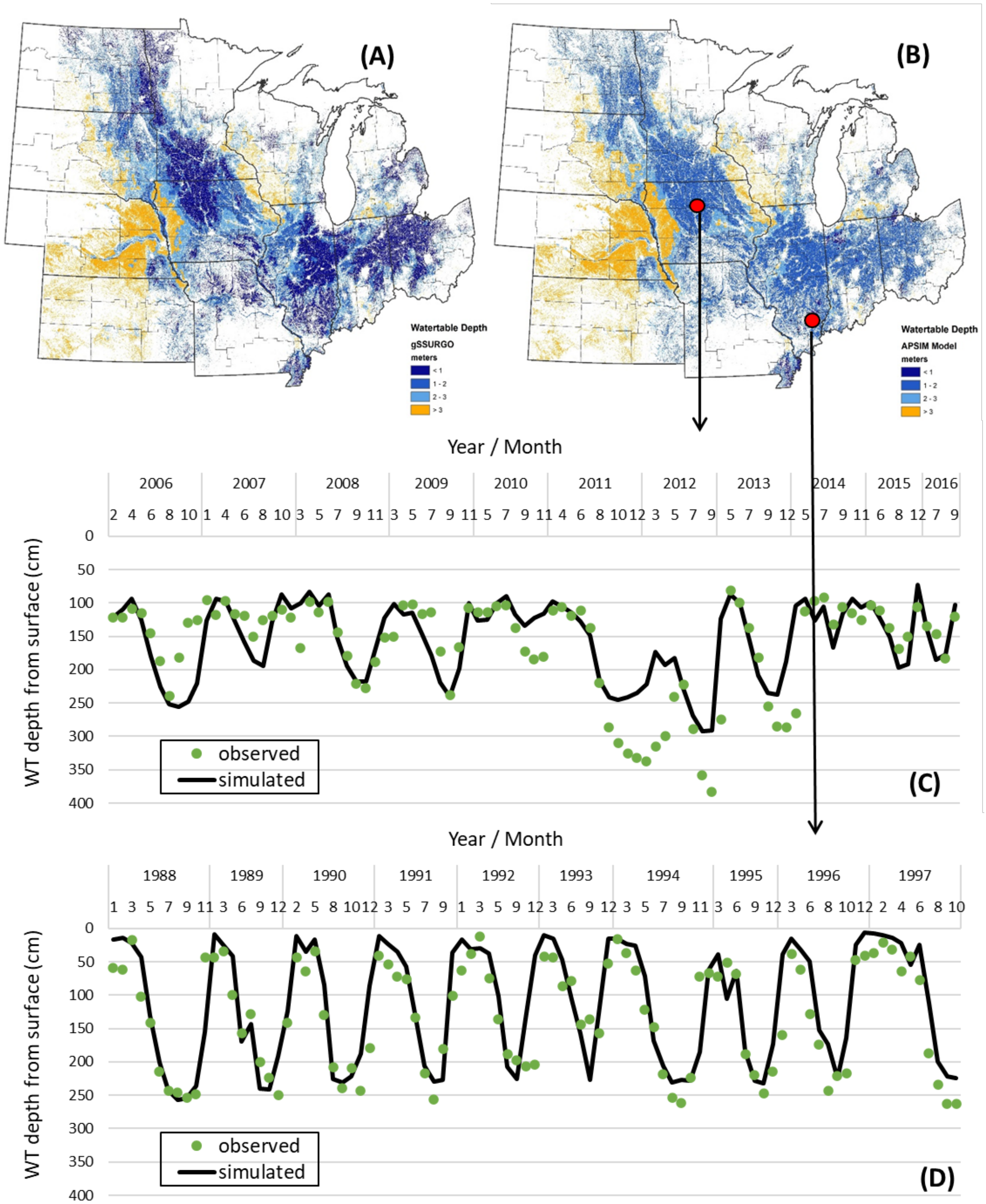

Fig. S5. gSSURGO water table depths in the US Corn Belt (panel A) and the Enhanced Crop Model simulated average water table depths (panel B) for the period 1984 to 2019. Temporal simulations against observational well data are shown for two locations (Kanawha, Iowa and Dixon Spring, Illinois). The well times series used in these figures is available at this paper's data repository (10.5281/zenodo.11393498); the original data source for the Kanawha location is Iowa State University and for Dixon Spring was provided by the Illinois State Water Survey (ISWS; https://isws.illinois.edu).

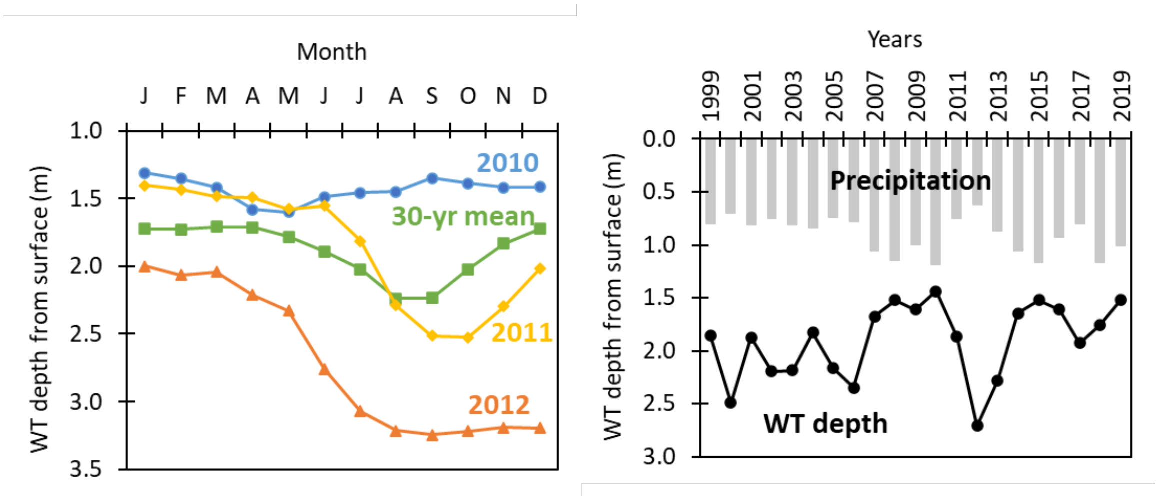

Fig. S6. Simulated water table depths by month (left) and by year using the Enhanced Crop Model for central Iowa Crop Reporting District.

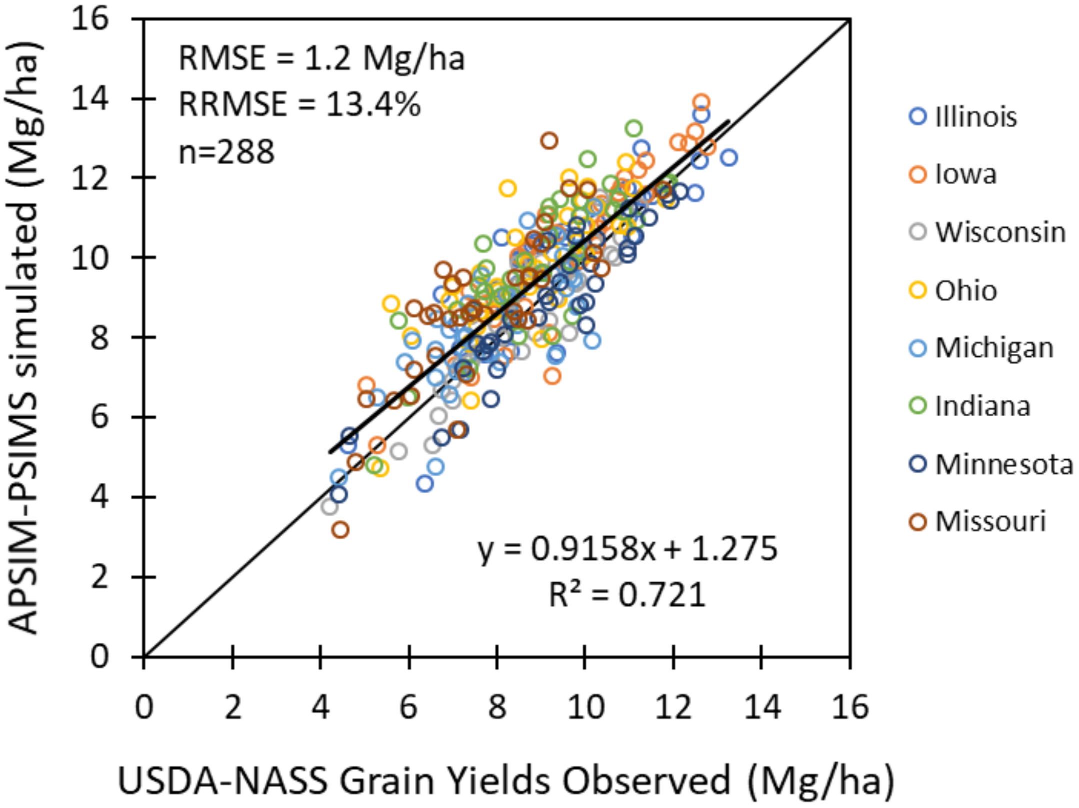

Fig. S7. Agreement between the Enhanced Crop Model simulated state-level maize grain yields versus USDA-NASS statistics for the period 1984 to 2019.

Table S1. Candidate variables and data sources. The full set of environmental covariables sampled at each location. gSSURGO = USDA Natural Resources Conservation Service Soil Survey (1); GRIDMET = Gridded Surface Meteorological 4km resolution daily dataset (2).

Variable	Time Aggregation	Source
Slope	Static	USGS NED
Soil root zone available water content	Static	SSURGO
Soil productivity index, corn	Static	SSURGO
Soil organic carbon (top 1 meter)	Static	SSURGO
Soil drainage class	Static	SSURGO
Early season precipitation	Annual; January - April	GRIDMET
Growing season precipitation	Annual; June - August	GRIDMET
Growing Degree Days (corn calculation)	Annual; January - August	GRIDMET - derived
Solar radiation	Annual; June - August	GRIDMET
April precipitation	Annual	GRIDMET
April mean maximum temperature	Annual	GRIDMET
April mean minimum temperature	Annual	GRIDMET
May precipitation	Annual	GRIDMET
May mean maximum temperature	Annual	GRIDMET
May mean minimum temperature	Annual	GRIDMET
June precipitation	Annual	GRIDMET
June mean maximum temperature	Annual	GRIDMET
June mean vapor pressure deficit	Annual	GRIDMET
June mean minimum temperature	Annual	GRIDMET
July precipitation	Annual	GRIDMET
July mean maximum temperature	Annual	GRIDMET
July mean minimum temperature	Annual	GRIDMET
July mean vapor pressure deficit	Annual	GRIDMET
July water deficit (Precip - PET)	Annual	GRIDMET - derived
August mean maximum temperature	Annual	GRIDMET
August mean minimum temperature	Annual	GRIDMET
August mean vapor pressure deficit	Annual	GRIDMET
August precipitation	Annual	GRIDMET
August water deficit (Precip - PET)	Annual	GRIDMET - derived
May water table depth	Annual	Enhanced Crop Model
June water table depth	Annual	Enhanced Crop Model
July water table depth	Annual	Enhanced Crop Model
August water table depth	Annual	Enhanced Crop Model
Annual water table depth	Annual	Enhanced Crop Model
Latitude	Static	
Longitude	Static	
Year	Annual	

Table S2. The full set of environmental covariables considered can be found in Table S1. gSSURGO = USDA Natural Resources Conservation Service Soil Survey (1); GRIDMET = Gridded Surface Meteorological 4km resolution daily dataset (2). We use the Standard Crop Model to provide July soil moisture to primarily capture soil moisture arising from non-groundwater sources.

Variable	Time Aggregation	Source
Soil root zone available water content	Static	gSSURGO
Soil productivity index, corn	Static	gSSURGO
Soil drainage class	Static	gSSURGO
July Soil Moisture (1 m)	Annual	Standard Crop Model
Growing season precipitation	Annual; June - August	GRIDMET
Solar radiation	Annual; June - August	GRIDMET
April mean minimum temperature	Annual	GRIDMET
May precipitation	Annual	GRIDMET
May mean maximum temperature	Annual	GRIDMET
June precipitation	Annual	GRIDMET
June mean vapor pressure deficit	Annual	GRIDMET
July precipitation	Annual	GRIDMET
July mean minimum temperature	Annual	GRIDMET
July mean vapor pressure deficit	Annual	GRIDMET
July water deficit (Precip - PET)	Annual	GRIDMET - derived
August mean maximum temperature	Annual	GRIDMET
August mean vapor pressure deficit	Annual	GRIDMET
July water table depth	Annual	Enhanced Crop Model
Year	Annual	

SI References

1. NRCS, SSURGO Web Soil Survey. *USDA Natural Recources Conservation Service* (2016).

2. J. T. Abatzoglou, Development of gridded surface meteorological data for ecological applications and modelling. *Int. J. Climatol.* **33**, 121–131 (2013).
